# Metabolic syndrome according to different definitions in a rapidly developing country of the African region

**DOI:** 10.1186/1475-2840-7-27

**Published:** 2008-09-18

**Authors:** Clara Kelliny, Julita William, Walter Riesen, Fred Paccaud, Pascal Bovet

**Affiliations:** 1 Institute of Social and Preventive Medicine (IUMSP), University Hospital Centre and University of Lausanne, rue du Bugnon 17, 1005 Lausanne, Switzerland; 2 Ministry of Health and Social Development, Victoria, Republic of Seychelles; 3 Institute of Clinical Chemistry and Hematology, Canton Hospital, St Gallen, Switzerland

## Abstract

**Aims:**

We examined, in a country of the African region, i) the prevalence of the metabolic syndrome (MetS) according to three definitions (ATP, WHO and IDF); ii) the distribution of the MetS criteria; iii) the level of agreement between these three definitions and iv) we also examined these issues upon exclusion of people with diabetes.

**Methods:**

We conducted an examination survey on a sample representative of the general population aged 25–64 years in the Seychelles (Indian Ocean, African region), attended by 1255 participants (participation rate of 80.3%).

**Results:**

The prevalence of MetS increased markedly with age. According to the ATP, WHO and IDF definitions, the prevalence of MetS was, respectively, 24.0%, 25.0%, 25.1% in men and 32.2%, 24.6%, 35.4% in women. Approximately 80% of participants with diabetes also had MetS and the prevalence of MetS was approximately 7% lower upon exclusion of diabetic individuals. High blood pressure and adiposity were the criteria found most frequently among MetS holders irrespective of the MetS definitions. Among people with MetS based on any of the three definitions, 78% met both ATP and IDF criteria, 67% both WHO and IDF criteria, 54% both WHO and ATP criteria and only 37% met all three definitions.

**Conclusion:**

We identified a high prevalence of MetS in this population in epidemiological transition. The prevalence of MetS decreased by approximately 32% upon exclusion of persons with diabetes. Because of limited agreement between the MetS definitions, the fairly similar proportions of MetS based on any of the three MetS definitions classified, to a substantial extent, different subjects as having MetS.

## Introduction

The metabolic syndrome (MetS) represents a cluster of metabolic risk factors that co-occur to a greater degree than predicted by chance. While it is clear that MetS is associated with cardiovascular disease and diabetes [1], it is still controversial whether MetS adds predictive value that goes beyond that of its single constituents [1,2]. Insulin resistance was initially the main focus of MetS, but adiposity, sedentary lifestyle, dietary and genetic factors have also received much attention when considering the pathogenesis of MetS [3]. High prevalence of MetS has often been documented in developed countries and increasingly so in developing countries [4-17], although data in developing countries and particularly in the Sub-Saharan region remain scarce [18].

Over the past decade, several definitions of MetS have been coined. In this paper, we consider three major definitions: i) the definition of the World Health Organization (WHO), issued in 1998 [19]; ii) the definition of the National Cholesterol Education Program Adult Treatment Panel III (ATP), issued in 2001 and updated in 2004 and 2005 [20], and the definition of the International Diabetes Federation (IDF), introduced in 2005 [21]. These definitions agree that the core criteria of MetS include: i) blood glucose impairment (hyperglycemia and/or insulin resistance), ii) excess abdominal/body fat (increased waist and/or obesity), iii) dyslipidemia (low HDL-cholesterol and/or high triglycerides), and iv) elevated blood pressure. However, criteria and cut-off values differ between these definitions, implying that different definitions may identify different people, as documented in the few studies that have addressed this question [4,7,8,22].

There is some controversy over whether identification of MetS should exclude people with diabetes since diabetes alone is sufficient to define high cardiovascular risk and MetS has been used as a tool to predict diabetes [2,23].

This analysis had four main objectives. First, we evaluated the prevalence of MetS according to three MetS definitions in the Seychelles, a rapidly developing country of the African region. Second, we examined the distribution of the MetS criteria according to the different MetS definitions. Third, we compared the level of agreement between the different MetS definitions and their ability to identify the same subjects. Fourth, we examined how the prevalence of MetS and the other end points differed upon restricting assessment of the metabolic syndrome to the non-diabetic population.

## Methods

### Survey procedures

The Republic of Seychelles is a group of islands in the Indian Ocean (African region) situated approximately 1800 km east of Kenya. A large majority of the population is of African descent. HIV and cardiovascular diseases account for approximately 1% and 38% of total mortality, respectively [24]. High prevalence of several cardiovascular risk factors including obesity, hypertension and diabetes has been documented as early as in 1989 [25] and in 2004 [26-28].

A population-based survey of cardiovascular risk factors was conducted in 2004 under the auspices of the Ministry of Health of the Republic of Seychelles (Seychelles Heart Study III). Methods and main results have been published previously [26-28]. Briefly, the sampling frame consisted of a sex and age stratified random sample of the entire population aged 25–64 years. Eligible participants were selected from computerized data of a national population census in 2002, thereafter updated by the civil status authorities. The survey was approved by the Ministry of Health after technical and ethical reviews. Eligible participants were invited by a letter to attend the survey at study centers on specified dates. They were free to participate and gave written informed consent.

Blood pressure was defined as the average of the last two of three measurements with a mercury sphygmomanometer taken at intervals longer than 2 minutes after the participants had been sitting for at least 30 minutes. Weight was measured with precision electronic scales (Seca, Hamburg) and height was measured with a fixed stadiometer.

Fasting blood was taken between 7:00 and 10:00 am. Serum was obtained within 2 hours of blood collection and immediately frozen to -20°C. Blood lipids were measured with standard methods. Fasting blood glucose (FBG) was analyzed immediately with a point-of-care analyzer (Cholestec LDX, Hayward, USA). If glucose was ≥ 5.6 mmol/l and the participant was not aware of having diabetes, an additional capillary measurement was performed within 10 minutes with a glucometer that adjusts readings to plasma values (Ascencia Elite, Bayer) and the average of the two readings was considered [27]. An oral glucose tolerance test (OGTT) was performed on all people who had FBG between 5.6 and 7.0 mmol/L but had never been diagnosed with diabetes. Fasting serum insulin was measured using commercial RIA kits (LINCO Research Inc, Missouri, USA). Microalbuminuria was measured with a semi-quantitative method using a Clinitek Status analyzer (Bayer, Leverkussen, Germany) [28].

### Definitions of the metabolic syndrome

The prevalence of MetS was calculated according to the standard criteria detailed in Table 1. For the WHO definition, we defined insulin resistance as the upper quartile of the homeostasis model assessment of insulin resistance (HOMA-IR), calculated as [fasting serum insulin level (microU/ml) × fasting blood glucose level (mmol/L)]/22.5 [29], which has been shown to be a reliable estimate of insulin resistance both among diabetic and non diabetic subjects [30,31]. For the IDF definition, we used ethnic-specific cutoff values for waist circumference, i.e. the same criteria for Europid and Sub-Saharan subjects (≥ 94 cm for men and ≥ 80 cm for women) [21] and, by extension, for people of 'mixed' descent, and ≥ 90 cm for men and ≥ 80 cm for women of the 4% of participants considered as 'Indian' or 'Chinese'. Information on treatment for dyslipidemia was not available at an individual level and we assumed that no one was under treatment, consistent with the very low number of people treated in Seychelles for dyslipidemia.

**Table 1:** Criteria for three definitions of the metabolic syndrome (WHO, ATP, IDF)

	**WHO 1998**	**NCEP ATP III 2005**	**IDF 2005**
	Diabetes, IFG, IGT or insulin resistance^1^ *plus *two or more of the remaining criteria.	Any three or more criteria.	Central obesity *plus *two or more of the remaining criteria.
1. Adiposity	Waist/hip ratio > 0.9 (M), > 0.85 (F) or BMI > 30 kg/m^2^	WC ≥ 102 cm (M), ≥ 88 cm (F)	WC ≥ 94 cm (M), ≥ 80 cm (F)^2^
2. Raised blood pressure	BP ≥ 140/90 mmHg or medication	BP ≥ 130/85 mmHg or medication	
3. Dyslipidemia	TG ≥ 1.7 mmol/l or HDL < 0.9 mmol/L (M), < 1.0 mmol/L (F)	TG ≥ 1.7 mmol/L or medication^3^	
		HDL-C < 1.03 mmol/L (M) or < 1.29 mmol/L (F) or medication^3^	
4. Impaired glucose regulation (dysglycemia)	Diabetes, IFG (FBG ≥ 6.1 mmol/l), IGT, or insulin resistance^1^	FBG ≥ 5.6 mmol/L^4^	FBG ≥ 5.6 mmol/L or previously diagnosed diabetes
5. Microalbuminuria	Microalbuminuria: albumin ≥ 20 μg/min or albumin/creatinine ratio (ACR) ≥ 30 mg/g		

WHO: World Health Organization [19]; ATP: National Cholesterol Education Program-Adult Treatment Panel III [20]; IDF: International Diabetes Federation [21].

BMI: body mass index; BP: blood pressure; HDL-C: high density lipoprotein cholesterol; FBG: fasting blood glucose; IFG: impaired fasting glucose; IGT: impaired glucose tolerance; TG: triglycerides; WC: waist circumference.

^1 ^Glucose uptake below lowest quartile under hyperinsulinemic euglycemic conditions for background population under investigation.

^2 ^Cut off for Sub Saharan Africans, Europids, and Eastern Mediterranean and Middle Eastern populations; ≥ 90 cm (M), ≥ 80 cm (F) for South Asians, Chinese, Japanese, and ethnic South and Central Americans [21].

^3^Specific treatment for hypertriglyceridemia or low HDL-cholesterol such as fibrate or nicotinic acid.

^4^This cut off was lowered from ≥ 6.1 mmol/L to ≥ 5.6 mmol/l in 2005 [20].

### Analysis

Analysis was performed with Stata 9.0. All analyses were standardized for age using the new WHO standard population [32]. Agreement between the three MetS definitions was determined using the Kappa statistic (κ). We graphed Venn diagrams for agreement between different definitions of MetS using the MATLAB 7.6 software, which allows representing areas proportionally to the corresponding numbers of cases. P values < 0.05 were considered statistically significant.

## Results

### Characteristics of the population

From the 1563 eligible participants, 1255 participated in the survey (80.3% participation rate) and 1218 had all necessary measurements to evaluate MetS according the three considered definitions and were included in this study. The distribution of selected risk factors and MetS criteria is presented in Table 2.

**Table 2:** Age-standardized distribution of selected risk factors in the population aged 25–64 years

		All	SD	Men	SD	Women	SD
N		1218		548		679	
Age (years)		42.0	10.8	42.1	10.7	41.9	11.0
Anthropometric parameters							
BMI (kg/m^2^)		26.9	5.7	25.5	4.7	28.3	6.3
Waist circumference (cm)		89.5	13.0	88.9	11.9	90.2	14.1
Adiposity ATP (%)	WC ≥ 102 cm (M), ≥ 88 cm (W)	35.2		14.0		56.0	
	WHR > 0.9 (M), > 0.85 (W) or BMI	61.5		50.7		72.1	
Adiposity WHO (%)	> 30						
Adiposity IDF (%)	WC ≥ 94 cm (M), ≥ 80 cm (W)	55.8		35.8		75.6	
Blood pressure							
Systolic BP (mmHg)		127.7	18.6	131.0	17.7	124.4	18.8
Diastolic BP (mmHg)		83.3	11.8	85.5	11.7	81.2	11.5
Raised BP – ATP/IDF (%)	BP ≥ 130/85 mmHg or medication	51.7		57.0		46.4	
Raised BP – WHO (%)	BP ≥ 140/90 mmHg or medication	39.5		43.3		35.7	
Glucose regulation							
Fasting glucose (mmol/L)		5.9	2.0	6.0	2.1	5.7	1.8
Insulin (pmol/L)		14.8	12.1	13.6	11.6	16.1	12.4
Impaired fasting glucose (%)		14.1		20.5		7.7	
Impaired glucose tolerance (%)		12.6		13.9		11.2	
Diabetes (%)		10.7		9.7		11.7	
Dysglycemia ATP (%)	FBG ≥ 5.6 mmol/l	33.3		40.0		26.7	
Dysglycemia WHO (%)	DM, IFG, IGT or insulin resistance	36.1		35.5		36.7	
Dysglycemia IDF (%)	FBG ≥ 5.6 mmol/l or history of DM	33.9		40.4		27.5	
Blood lipids							
Total cholesterol (mmol/L)		5.4	1.3	5.4	1.3	5.4	1.3
HDL cholesterol (mmol/L)		1.4	0.5	1.4	0.5	1.4	0.4
Triglyceride level (mmol/L)		1.0	0.8	1.2	1.0	0.9	0.5
Hypertriglyceridemia ATP/IDF (%)	TG ≥ 1.7 mmol/l or medication	12.6		17.4		7.8	
Low HDL-C ATP/IDF (%)	HDLC < 1.03 (M); < 1.29 (W)	38.2		28.6		47.8	
Dyslipidemia WHO (%)	TG ≥ 1.7 or HDL < 0.9 (M), < 1.0 (W))	23.8		27.3		20.3	
Microalbuminuria		11.9		11.2		12.6	
Regular cigarette smoking (%)		17.3		30.9		3.7	
Alcohol (mL/day)		22.3	62.0	41.1	82.8	3.6	12.8

Mean ± SD (standard deviation) or proportion; BMI: body mass index; WC: waist circumference; WHR: waist to hip ratio; BP: blood pressure; DM: diabetes; IFG: impaired fasting glucose; IGT: impaired glucose tolerance; M: men; W: women. HDL-cholesterol and triglyceride: units in mmol/L.

### Prevalence of the metabolic syndrome

**Figure 1:**
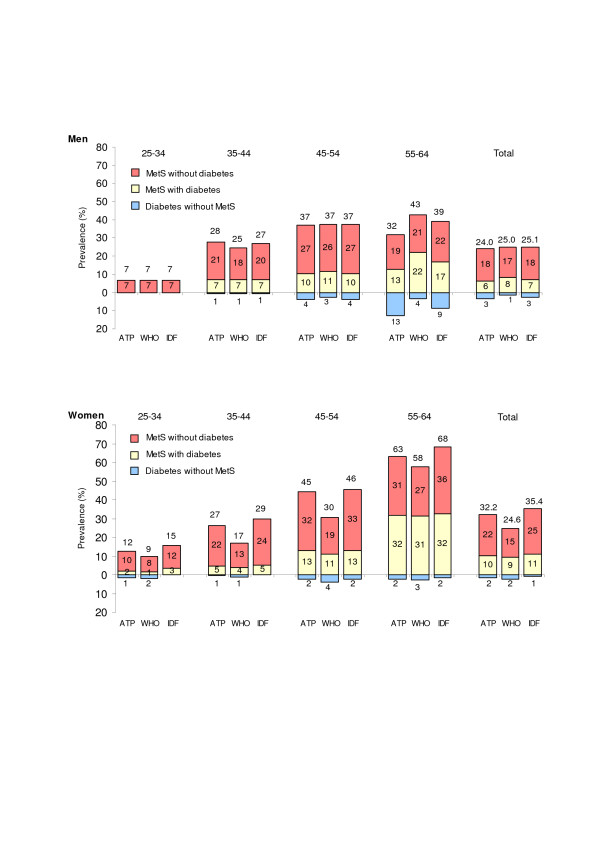
Prevalence of the metabolic syndrome according to sex, age category, diabetic status and different definitions of the metabolic syndrome.

Figure 1 shows the prevalence of MetS according to the definitions by ATP, WHO and IDF. The age-standardized prevalence of MetS in the population aged 25–64 years was 28.1% (95% CI: 25.6–30.7), 24.8% (22.4–27.2) and 30.3% (27.7–32.9), respectively. At age 35–64, the age-standardized prevalence was, respectively, 36.7% (33.6–39.7), 32.4% (29.5–35.4) and 39% (35.9–42.1) overall; 31.9% (27.5–36.4), 33.4% (28.9–37.9) and 33.5% (29.0–38.0) among men; and 41.4% (37.1–45.6), 31.4% (27.5–35.4) and 44.4% (40.2–48.7), among women.

The prevalence of MetS increased markedly with age for both genders. However, the prevalence of MetS using the ATP definition was highest at age 45–54 for men. With regards to gender, the prevalence was significantly greater in women than in men according to the ATP and IDF definitions while the prevalence was similar in both genders using the WHO definition.

A large proportion of people with diabetes also had MetS: 66.8%, 85.5%, 74% among men and 87.1%, 79.7%, 93% among women, according to the ATP, WHO and IDF definitions, respectively. However, this proportion was smaller among men at age 45–64 years than in the other age categories (Figure 1).

Upon exclusion of individuals with diabetes, the age-standardized prevalence of MetS at age 25–64 years was 22.2% (19.6–24.7) according to ATP, 17.9% (15.6–20.2) according to WHO and 23.8% (21.2–26.4) according to IDF. Using the EGIR definition of MetS [33], a modified version of the WHO definition that applies to non-diabetic subjects, the age-standardized prevalence of MetS was 15.1% (95% CI: 12.9–17.3); 14.1% (10.9–17.2) among men and 16.2% (13.1–19.2) among women.

Upon exclusion of persons with diabetes, the prevalence (in absolute terms) of MetS decreased by 4.6%, 6.5%, 5.3% in men and 7.3%, 7.3%, 7.7% in women according to the ATP, WHO and IDF definitions, respectively. This corresponds to a relative decrease in the prevalence of MetS of approximately 32%. At the age of 35–64 years, the decrease in prevalence (in absolute terms) was respectively 5.8%, 8.6%, 6.7% in men and 7.8%, 6.7%, 8.8% in women (a relative decrease of approximately 34%). The reduction in prevalence of MetS upon exclusion of subjects with diabetes was slightly greater when using the WHO definition of MetS than the other two definitions.

### Distribution of criteria of the metabolic syndrome

**Figure 2:**
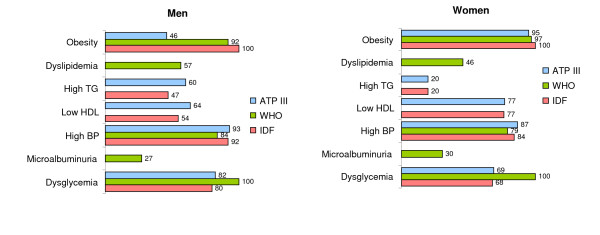
Proportion of subjects with the metabolic syndrome who have selected criteria according to sex and different definitions of the metabolic syndrome (TG: triglyceride; HDL: HDL-cholesterol; BP: blood pressure).

High blood pressure, obesity and impaired glucose regulation were the most prevalent criteria for all MetS definitions (Figure 2). Hypertriglyceridemia was found more often in men than in women, while the opposite was true for low HDL-cholesterol. This criteria distribution was similar across age categories.

### Agreement between different definitions of the metabolic syndrome

**Figure 3:**
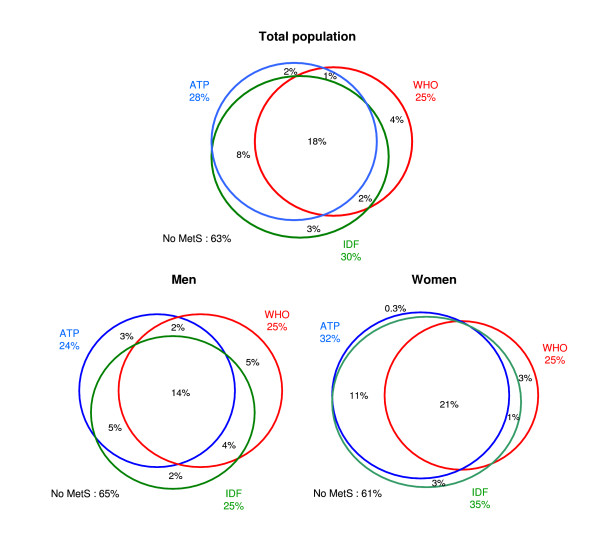
Agreement between three definitions of the metabolic syndrome (all prevalence estimates are expressed as percent of the general population).

Figure 3 shows the level of agreement between the three MetS definitions. 37% of all subjects aged 25–64 had MetS as based on any of the three MetS definitions. Among these MetS-holders, 69% had MetS based on ATP and IDF, 50% based on ATP and WHO, 53% based on WHO and IDF; and 48% had MetS based on the three definitions. With reference to the total population, MetS was diagnosed in 28% based on ATP, 25% based on WHO and 30% based on IDF. Based on Figure 3, one can calculate that 25% were diagnosed with MetS based on ATP and IDF, 20% based on WHO and IDF, 19% based on WHO and ATP; and 18% based on all three definitions.

Within the non-diabetic population, 30% had MetS based on either the WHO, ATP or IDF definitions, out of which 37% had MetS based on all three definitions.

The kappa statistic was 0.82 for ATP-IDF, 0.61 for IDF-WHO and 0.59 for WHO-ATP, respectively 0.81, 0.53 and 0.51, upon exclusion of people with diabetes. The higher kappa statistic for ATP-IDF versus either IDF-WHO or WHO-ATP was found irrespective of gender and age (Table 3).

**Table 3:** Agreement (kappa values) between different definitions of the metabolic syndrome in the population with and without exclusion of diabetic persons

	ATP-IDF		IDF-WHO		WHO-ATP	
	All	Non-diabetic	All	Non-diabetic	All	Non-diabetic

All	0.82	0.81	0.61	0.53	0.59	0.51
Men	0.71	0.67	0.62	0.55	0.56	0.48
Women	0.92	0.91	0.60	0.52	0.63	0.53
Age 25–44 years	0.79	0.76	0.49	0.42	0.52	0.44
Age 45–64 years	0.83	0.83	0.64	0.58	0.58	0.53

In order to examine possible causes of fairly low agreement between the MetS definitions, we compared agreement between the criteria of the three MetS definitions in the entire population (Table 4). The largest disagreement was found for adiposity. As many as 70% of the total population fulfilled the criteria for adiposity according to either the ATP, WHO or IDF definitions of MetS (i.e. based on BMI, waist circumference or waist to hip ratio). However, the prevalence of the adiposity criterion varied largely according to the different MetS definitions: 35.2% according to ATP, 61.5% according to WHO, and 55.8% according to IDF. Out of those who qualified for adiposity according to any MetS definitions, only 48% met all three definitions. With regards to the other MetS criteria, the WHO definition disagreed with the other two definitions of MetS (ATP and IDF) in identifying people with dyslipidemia and to a lesser extent, with impaired glucose regulation. However, these criteria are less prevalent in the population than adiposity (Table 2), which results in less impact on MetS classification.

**Table 4:** Kappa values between different criteria of different definitions of the metabolic syndrome

	ATP-IDF	IDF-WHO	WHO-ATP
***Total population***			
Obesity	0.60	0.55	0.44
Raised blood pressure	1.00	0.76	0.76
Impaired glucose regulation	0.99	0.67	0.66
Hypertriglyceridemia	1.00	1.00	1.00
Low HDL-cholesterol	1.00	0.50	0.50
***Men***			
Obesity	0.45	0.55	0.27
Raised blood pressure	1.00	0.73	0.73
Impaired glucose regulation	0.99	0.65	0.64
Hypertriglyceridemia	1.00	1.00	1.00
Low HDL-cholesterol	1.00	0.69	0.69
***Women***			
Obesity	0.58	0.45	0.50
Raised blood pressure	1.00	0.78	0.78
Impaired glucose regulation	0.98	0.70	0.68
Hypertriglyceridemia	1.00	1.00	1.00
Low HDL-cholesterol	1.00	0.36	0.36

## Discussion

The main findings of the study are as follows: i) the prevalence of MetS was high in this population of East Africa regardless of which MetS definition was used; ii) the prevalence of MetS decreased by approximately one third upon exclusion of persons with diabetes; and iii) agreement between different MetS definitions was limited and consequently, the similar prevalence of MetS according to either MetS definition actually identified, to a substantial extent, different subjects as having MetS.

In our study, the prevalence of MetS at the age of 25–64 years ranged between 25% and 30%. This is much higher than the 8% reported in Cameroon [18], which is, to the best of our knowledge, the only other population based published assessment of the prevalence of MetS in Sub-Saharan Africa. A previous study in the Seychelles [34] found that plasma aldosterone, but not plasma renin activity, was associated with MetS. However this study included participants from families with hypertension and was therefore not intended to assess the prevalence of MetS in the general population. In order to compare the prevalence in Seychelles with that in other countries, we compiled findings of selected population-based studies that had assessed MetS according to at least two MetS definitions, and included participants of 35–64 years (Table 5). The prevalence of MetS was similar in Seychelles as in many Western countries [9-11,13] and in urban India [15]. The prevalence of MetS in Seychelles was lower than in certain countries, e.g. USA [5], Portugal [8], Samoa [13], Turkey [7] and Tunisia [4] but higher than in Mexico [6] and several Asian countries e.g. Korea [14], Japan [13] and China [17]. The high prevalence of MetS in Seychelles is consistent with high prevalence of several MetS criteria, particularly overweight, hypertension, dyslipidemia and diabetes [25-27].

**Table 5:** Prevalence of the metabolic syndrome in the Seychelles and in other populations, according to different definitions of the metabolic syndrome

				ATP			WHO			IDF		
				
Place	Population	Age	n	M	W	All	M	W	All	M	W	All
***Africa***												
Seychelles	Nation wide	25–64	1218	24	32	28	25	25	25	25	35	30
Tunisia [3]	City of Tunis	≥ 40	863	15	31	24	26	31	29	30	56	46
Cameroon[17]	City of Yaoundé and three rural villages	24–74	1573	< 0.5	< 0.2	-	< 8	< 6	-	< 2	< 2	-
***Americas***												
USA [4]	NHANES 1999–2002	≥ 20	3601	34	35	35	-	-	-	41	37	39
Mexico [5]	Nation wide	20–69	2158	29	25	27	13	14	14	-	-	-
***Europe***												
Turkey [6]	Istanbul (urban) and Kayseri (rural)	> 20	1568	41	43	38	23	18	19	46	48	42
Portugal [7]	City of Porto	18–92	1433	32	40	37	30	24	26	38	44	42
Canary Island [8]	Nation wide	> 30	1030	28	29	28	33	24	28	-	-	-
Norway [9]	North-Trondelag Health Study (HUNT 2)	20–89	10,206	27	25	26	-	-	-	29	30	30
Germany [10]	PROCAM study	16–65	7131	25	18	-	-	-	-	32	23	-
Greece [11]	Representative sample of Greek adults	> 18	9,669	25	24	25	-	-	-	-	-	43
***Asia***												
Samoa [12]	Population-based (DETECT 2)	> 35	1344	39	57	-	22	26	-	45	60	-
Australia [12]	Population-based (DETECT 2)	> 35	9409	36	28	-	26	18	-	42	33	-
Korea [13]	Korean National Health and Nutrition Survey	≥ 20	6601	18	21	19	-	-	-	15	24	20
India [14]	City of Chennai	≥ 20	2350	17	19	18	27	20	23	23	28	26
Taiwan [15]	Taiwan National Nutrition and Health Survey	≥ 19	2608	12	17	-	-	-	-	6	13	-
Japan [12]	Population-based (DETECT 2)	> 35	2016	8	10	-	3	3	-	8	11	-
China [16]	Two agricultural counties	25–64	18,630	5	12	8	-	-	-	4	11	7

Men: men; W: women, ATP: National Cholesterol Education Program-Adult Treatment Panel III; WHO: World Health Organization, IDF: International Diabetes Federation.

The prevalence of MetS in Seychelles did not differ markedly according to the three different MetS definitions, consistent with observations in several populations [5,9,10,14,17], but not in others [4-7,12,13]. However, the prevalence of MetS in Seychelles was moderately higher according to IDF than ATP or WHO, a finding reported in most studies that had assessed this issue [4,5,7,8,10-13,15], except for a few [16,17]. It has frequently been reported that the prevalence of MetS according to the WHO definition is generally greater among men than women [35] but we did not observe such a gender difference in Seychelles.

The level of agreement between the different definitions of MetS was not optimal in our study. Less than half of the individuals labeled as having MetS according to any of the three considered definitions were classified as MetS-holders according to all three definitions. We found that agreement between the MetS definitions was better for IDF-ATP than for WHO-IDF and WHO-ATP. This finding is consistent with data in several populations on several continents [7,8,10,13,15]. This difference is expected since MetS is based on the same criteria according to IDF and ATP, except for the adiposity criterion. Agreement between the MetS definitions was generally better among women than men in Seychelles, consistent with previous reports [5,8,14]. This may relate to the fact, at least in Seychelles, that the prevalence of the adiposity criterion was virtually identical and close to 100% across the three MetS definitions among women with MetS, but was lower and largely different between MetS definitions in men.

Hence, we found a similar prevalence of MetS in Seychelles according to the three different MetS definitions but the different MetS definitions actually identified, to a substantial extent, different individuals. Poor agreement between MetS definitions has several clinical and epidemiological implications [16,22]. First, it is questionable to directly compare the burden of MetS between populations based on different definitions. Second, it remains unclear whether cardiometabolic outcomes differ if MetS is defined according to one, two or three MetS definitions. Few studies have assessed the predictive value of MetS when MetS is based on more than one definition [36]. The study of Benetos et al [36] showed that the prevalence of MetS was markedly higher when MetS was based on either the IDF or ATP (2005 version) definitions as compared to the 2001 ATP definition alone, but only individuals with MetS according to the 2001 ATP definition had a higher risk of all-cause and cardiovascular mortality.

The prevalence of MetS restricted to the non-diabetic population (e.g. according to the EGIR definition, which is a definition of MetS that explicitly excludes diabetes [33]) was similar in Seychelles as in several countries in Europe [37-39] and higher than in Japan and Korea [13]. In the Seychelles, about one third of the adults who had MetS also had diabetes and, inversely, most adults who had diabetes also had MetS. Hence, the prevalence of MetS, according to either the ATP, WHO and IDF definitions, decreased markedly (a relative decrease of approximately one third) upon exclusion of persons with diabetes. This difference is larger than reported in other studies [6,13]. We also observed that this decrease in the prevalence of MetS upon exclusion of diabetes was larger according to the WHO definition compared to the ATP and IDF definitions, which has also been observed in Samoa [13] and Mexico [6].

The issue of whether MetS should be defined upon exclusion of diabetes is relevant to both clinical practice and epidemiology. It has been argued that MetS status does not add incremental information for cardiovascular management of diabetic individuals [23], since diabetes alone defines high cardiovascular risk. For the same reason, it has been suggested that the definition of MetS should exclude individuals with known cardiovascular disease [23]. The question of whether MetS should exclude diabetes and/or cardiovascular disease is clearly dependent on the expected purpose of MetS, i.e. whether MetS is used to predict cardiovascular diseases, diabetes, insulin resistance, or a combination of these conditions independent of conventional risk factors. While our cross-sectional study emphasizes that different definitions of MetS tend to classify different individuals as having MetS, the most important question remains to determine, based on longitudinal data, whether MetS predicts cardiovascular events above the risk factors that constitute the syndrome.

Our study has some limitations. First, we cannot exclude a healthy volunteer bias related to voluntary participation to the study, which could tend to underestimate the actual prevalence of MetS in the population. Second, we assessed insulin resistance based on HOMA-IR, which is only partially correlated with the gold standard (hyperinsulinemic euglycemic clamp), yet a valid proxy for insulin resistance [30,31]. Third, OGTT was only performed on participants unaware of having diabetes and who had FBG between 5.6 and 7.0 mmol/l, hence a source of slight underestimation of diabetes. On the other hand, strengths of the study include the population-based design of the study, a fairly large sample size, and a broad panel of investigations that allowed us to directly compare the different MetS definitions.

In conclusion, this study contributes to mapping the prevalence of MetS worldwide, particularly with regards to the African region. The study also further contributes to the longstanding debate regarding the significance of MetS. The substantially different prevalence of MetS upon exclusion of individuals with diabetes underlies the need to clarify whether MetS is a tool for predicting cardiovascular disease, diabetes, insulin resistance or any other risk condition. These issues are further prompted by the finding, in our study, that the different considered MetS definitions identified, to a substantial extent, different individuals. Not withstanding a much needed unified definition of MetS, our findings in Seychelles emphasize the growing burden of lifestyle-related non-communicable diseases in countries in epidemiological transition including in the African region, consistent with the ongoing epidemic of obesity worldwide and in the Seychelles in particular.

## List of abbreviations

ATP: National Cholesterol Education Program-Adult Treatment Panel III; WHO: World Health Organization; IDF: International Diabetes Federation; EGIR: European Group for the Study of Insulin Resistance; HDL-cholesterol: High density lipoprotein cholesterol; FBG: fasting blood glucose; HOMA-IR: homeostasis model assessment of insulin resistance; OGTT: oral glucose tolerance test.

## Competing interests

The authors declare that they have no competing interests.

## Authors' contributions

CK led the analysis of data and the write up of the manuscript; JW had a coordinating role in the conduct of the study and reviewed the manuscript; WR performed most of the blood analyses and reviewed the manuscript; FP participated in the study design and reviewed the manuscript; PB was the principal investigator of the study and actively participated in the data analysis and the write up of the manuscript.
